# Cladribine, cytarabine, and filgrastim based regimen in relapsed or refractory acute myeloid leukemia: A systematic review and meta-analysis

**DOI:** 10.1097/MD.0000000000034949

**Published:** 2023-11-03

**Authors:** Susu Cao, Qianshan Tao, Jia Wang, Qing Zhang, Yi Dong

**Affiliations:** a Department of Hematology, The Second Hospital of Anhui Medical University, Hefei, P.R. China; b Shaoxing People' Hospital, Shaoxing, Zhejiang, China.

**Keywords:** cladribine, cytarabine, filgrastim, meta analysis, relapsed or refractory AML

## Abstract

**Background:**

To ascertain the efficacy and safety of cladribine, cytarabine, and filgrastim-based regimen in relapsed or refractory (R/R) AML patients.

**Methods:**

Clinical studies were searched in PubMed, Cochrane Library, Embase data. We selected available factors including complete remission (CR), overall response rate (ORR), overall survival (OS) to evaluate the efficacy, and early death (ED), and adverse events to evaluate safety.

**Results:**

15 records with 812 R/R AML patients were finally included and analyzed using the R software. Subgroups analysis was also conducted. The pooled CR rate for CLAG regimen, CLAG-M regimen, and CLAG combined with any other drugs regimen is 56% (95% CI: 46–66), 46% (95% CI: 34–56), 44% (95% CI: 26–64), respectively. The relapsed and refractory groups showed a CR rate of 68% (95% CI: 53–80), and 51% (95% CI: 45–58) with CLAG related regimens. As risk grade decreases, the pooled CR rate increases. Regarding the safety for CLAG-related protocols, systematic review was conducted.

**Conclusion:**

The CLAG-related regimen is an effective and safe therapy for R/R AML patients, CLAG seems to have more superiority than CLAG combined therapy, though further studies including cladribine combination treatment protocols, are still needed to confirm our results further.

## 1. Introduction

Acute myeloid leukemia (AML) is a heterogeneous fatal disease featured by abnormal hematopoietic stem cells proliferation and differentiate suppression. Complete remission (CR) is only achieved in about 44% of younger (under the age of 55) newly diagnosed AML patients by the conventional intensive chemotherapy, and hematopoietic stem cell transplantation is considered as the only way to be cured.^[1–4]^ However, the situation is much more complex than we thought; nearly 10% to 40% younger patients and 40% to 60% older patients (above 60 years old) suffer primary refractory to induction chemotherapy. And 50% to 70% who obtain first CR still tend to be relapse; for those with complicated capabilities, severe physical reserve, response to therapy and duration of remission will also tend to be gloomy.^[5,6]^ According to some clinical studies, estimated overall survival (OS) did is not more than 10% in 3 years for relapsed or refractory AML (R/R AML), with estimated relapse rates high as 40% even after allo-hematopoietic stem cell transplantation treatment.^[5,7,8]^ Reported studies were indicated that R/R AML is an extremely aggressive form of disease and demonstrates some extent of remarkable malignant heterogeneity than AML itself. So salvage therapy for those patients urgently needs to be made out, or the future of those patients is definitely much poorer than it has been.

The CLAG regimen, compromised of cladribine, cytarabine, and filgrastim, is frequently reported in recent clinical studies, with findings showing support for effectiveness of R/R AML. CLAG regimens are also designed to be combined with other drugs such as imatinib mesylate (IM), mitoxantrone, aclarubicin, and seliniexor to improve the survival of patients with R/R AML. In recent years, many cohort studies described the efficacy of the CLAG or CLAG-combined regimen. The purpose of this systematic review and meta-analysis is to specifically analyze the efficacy and safety of CLAG related regimens for R/R AML patients.

## 2. Methods

### 2.1. Data sources and search

According to the principle described in the Cochrane Handbook for Systematic Reviews of Interventions, a systematic review and meta-analysis was conducted.^[9]^ We selected PubMed, Cochrane Library, Embase, these 3 databases for searching from January 2001 to March 2021 without language restriction. The references of included studies were also assessed. The keywords included “relapsed” or “relapse” or “resist” or “resistant,” “acute myeloid leukemia,” “cladribine” or “2-chlorodeoxyadenosine” or “2-CdA” or “leustatin.” The access to specific search strategy is available in supplementary data.

### 2.2. Inclusion and exclusion criteria

Inclusion criteria was presented as following: Adult patients diagnosed with R/R AML based on WHO criteria. R/R AML patients treated with CLAG-related protocol with sample size above 20. Original study was an randomized controlled trial (RCT), prospective cohort study, retrospective cohort study. CR rate was considered as the primary outcome. The Overall survival rate (ORR), median OS, early death (ED) rate were all regarded as secondary outcomes.

Exclusion criteria was presented as following: Study population other than adult R/R AML or sample size less than 20. Study type containing case report, review, or meta-analysis was also excluded. Treatment regimen other than CLAG-based regimen. Primary outcome data cannot be retrieved.

Two independent investigators assessed the eligibility, and differences were reviewed by both or with the other coauthors until consensus is reached.

### 2.3. Definition of treatment and treatment response and outcomes

Standard CLAG regimen consist of cladribine 5mg/m^2^/day d1 to 5; cytarabine 2g/m^2^/day d1 to 5; G-CSF 300µg/day d0 to 5. The CLAG-based regimen included a modified CLAG regimen or CLAG regimen combined with other drugs.

A patients assessed with CR should meet conditions of all the following: bone marrow blasts count below 5% at steady-state; without cytological evidence of leukemia; without transfusion requirement; without extramedullary invasion; a hemoglobin level above 11 g/dL; an absolute neutrophil count above 1.0 × 10^9^/L; and a platelet count of above 100 × 10^9^/L. Patients response considered as partial response (PR) should meet these conditions as following: Bone marrow blasts of 5% to 25% or a 50% or better decrease in BM blasts, or BM blasts below 5% but with Auer rods’ presence. CR plus PR is considered as ORR. An ED is defined as patients who died within 30 days in the treatment process. An OS is defined as the time from the disease diagnosis to the end of the follow-up or until patients dies.

### 2.4. Statistical analysis

All data analysis was performed using R software version 4.0.5. Data were extracted from the eligible studies according to the already designed table by 2 investigators. The incidence of CR, ORR, ED was combined with 95% confidence interval. Heterogeneity between studies was assessed using Cochran’s test and *I*^2^ statistics. The *I*^2^ values were classified into 4 different grades of heterogeneity as follows: insignificant heterogeneity (*I*^2^, 0–25%); low heterogeneity (*I*^2^, 26–50%); moderate heterogeneity (*I*^2^, 51–75%); high heterogeneity (*I*^2^, >75%). The Newcastle–Ottawa Scale criteria was used to evaluate quality of eligible studies. The complete scores are detailed in Table S1, Supplemental Digital Content, http://links.lww.com/MD/J846.^[10–24]^

## 3. Results

### 3.1. Baseline patients characteristics and the risk of bias

We searched 365 studies in PubMed, Cochrane Library, Embase database precluding 52 duplicates. 313 records were scanned through titles and abstracts, and 268 were excluded. Two investigators independently read the full text of 45 records, excluding studies containing ineligible patients sample size, non CLAG-based therapy regimen, and those without primary outcomes. Finally 15 eligible studies were selected for quality assessment, publication assessment, and data extraction according to a pre-designed stable. The process of selection is presented in the flow diagram (Figure S1, Supplemental Digital Content, http://links.lww.com/MD/J847).

All included eligible patients’ characteristics are completely described in Table S2, Supplemental Digital Content, http://links.lww.com/MD/J848. One of these studies is Chinese; the others are all English. There was a total of 771 R/R AML patients with 300 males. 307 R/R AML patients received a CLAG salvage regimen. 464 population group treated with CLAG combined with other drugs therapy. Two of these included studies are prospective studies, 8 are retrospective studies, 5 are phase I/II clinical trials. There were no RCTs that meet our eligibility criteria. Our meta-analysis restricted sample size values to above 20 with controlled variability for the sample size to some extent. The sample size for included studies ranged from 27 to 114, and the median age ranged from 45 to 63 years. 15 studies all reported CR, with 5 reported ORR, 8 reported ED. Median time and corresponding 95% CI of OS were available in 13 studies. All included trials all used cladribine, cytarabine, filgrastim containing treatment protocols to treat R/R AML patients, 9 of them combined CLAG with other drugs (Table S2, Supplemental Digital Content, http://links.lww.com/MD/J848).

We used the Newcastle-Ottawa Scale to evaluate all these eligible studies. Publication bias was assessed by funnel plots, as shown in Figure S2, Supplemental Digital Content, http://links.lww.com/MD/J849. There appears to have been a little publication bias in our included studies.

### 3.2. Efficacy

CR was available in all 15 studies, including 771 patients. CR rate ranged from 26% to 79%, the lowest CR rate was in the CLAG regimen combined with imatinib, and the highest was achieved on the standard CLAG regimen. The pooled CR rate for the whole study was 50% (95% CI: 43–58) with moderate heterogeneity (*I*^2^ = 73%, *t*^2^ = 0.2635, *P* < .01, Figure S3, Supplemental Digital Content, http://links.lww.com/MD/J850), so the result was determined by a random-effects model.

The pooled ORR for the 5 studies with 239 patients was 64% (95% CI: 49–76). ORR ranged from 38% to 78.7%, with the highest and lowest one respectively in the standard CLAG and the CLAG combined with imatinib protocol. However, a high heterogeneity existed between the studies (*I*^2^ = 81%, *t*^2^ = 0.3768, *P* < .01, Figure S4, Supplemental Digital Content, http://links.lww.com/MD/J851), thus finally, the random-effects model was used.

A descriptive systematic review of all included studies observed median OS in the range of 7.3 to 14 months. The median OS in the standard CLAG group was 7.3 to 14 months, in the CLAG-combined group 7.8 to 13.3 months. However, the study by Ye et al^[21]^ is a modified CLAG regimen without any drug combinations, with the median OS of the modified CLAG regimen was 10.8 months with 95% CI of 6.0–15.7.

### 3.3. Subgroup analysis

We did subgroup analysis according to different treatment protocols to explore the heterogeneity of the eligible studies. The number of CLAG combined with mitoxantrone was large enough for conducting a meta-analysis, so we regarded a CLAG-M therapy as a single subgroup. The results showed that the pooled CR rate of standard CLAG regimen was 56% (95% CI: 46–66), CLAG-M regimen was 46% (95% CI: 34–56), CLAG combined with other drugs regimen was 44% (95% CI: 26–64) which suggested that standard CLAG has a higher CR rate than CLAG based regimen. However, there was still high heterogeneity within these studies, so both subgroups were analyzed by the random-effects model (Figure S5, Supplemental Digital Content, http://links.lww.com/MD/J852).

We also conducted subgroups analysis according to disease type; it is showed that the CR rate of relapsed AML group was 68% (95% CI: 53–80), and the refractory AML group was 51% (95% CI: 45–58). Thus, it suggests that the CLAG-related therapy for relapsed patients group has numerical superiority over the refractory group. However, we observed different heterogeneity in these 2 groups; the relapsed group had moderate heterogeneity, while no heterogeneity existed in the refractory group (*I*^2^ = 71%, *t*^2^ = 0.5771, *P* < .01; *I*^2^ = 0, *t*^2^ = 0, *P* = .84) (Figure S6, Supplemental Digital Content, http://links.lww.com/MD/J853).

And we also conducted subgroup analysis to correlate different risk classifications for 7 eligible studies that described the CR rate of different risk classifications. The classifications were good risk for CR rate of 92% (95% CI: 78–97) with no heterogeneity (*I*^2^ = 0, *t*^2^ = 0, *P* = 1.00), CR rate for intermediate group was 62% (95% CI: 54–68) with insignificant heterogeneity (*I*^2^ = 0, *t*^2^ = 0, *P* = .54), and poor group was 43% (95% CI: 34–53) carrying low heterogeneity (*I*^2^ = 26%, *t*^2^ = 0.1656, *P* = .22) (Figure S7, Supplemental Digital Content, http://links.lww.com/MD/J854).

In addition, we as well as conducted a subgroup analysis correlated with different types of AML. There were only 3 studies reported CR rate of primary and secondary AML patients. The CR rate for primary AML patients was 67% (95% CI: 59–75) with low heterogeneity (*I*^2^ = 0%, *t*^2^ = 0.0, *P* = .57) and CR rate for secondary AML patients was 30% (95% CI: 16–49) also with low heterogeneity (*I*^2^ = 0%, *t*^2^ = 0.0, *P* = .70) (Figure S8, Supplemental Digital Content, http://links.lww.com/MD/J855).

### 3.4. Safety

Concerning the variety of toxicity within the included studies, we did a systematic review for CLAG-related regimens.

For adverse events for all the CLAG-related protocols, the most common adverse events reported during treatment was hematological toxicity, including neutropenia, and thrombocytopenia. In the CLAG group, almost all the patients experienced hematological toxicities.^[11,13]^ For the non-hematological adverse event, infections can existed in various body parts, particularly lung infection.^[11]^

The early death (ED) rate acquired from 7 studies was 9% (95% CI: 7–13), including 445 patients detected by the fixed-effect model, the heterogeneity was almost insignificant (*I*^2^ = 24%, *t*^2^ = 0.0276, *P* = .25, Figure S9, Supplemental Digital Content, http://links.lww.com/MD/J856). The ED rate for analyzed for available groups ranged from 5.3% to 17%. The CLAG combined with the imatinib group had the highest ED rate, while the lowest rate was observed in the standard CLAG regimen group.

### 3.5. Sensitivity analysis

Through removing studies by turn, the pooled CR rate ranged from 49% to 52% without large changes. It indicates that the results from our search are relatively stable and receivable.

## 4. Discussion

Early in 1996, researchers had proven that cladribine could increase the sensitivity of cytarabine and may be more effective when combined with filgrastim from studies in vivo or in vitro.^[25,26]^ Chow et al^[27]^ also reported that the combination of cladribine with cytarabine exhibited a potential of inhibiting myeloid leukemic cell proliferation, inducing apoptosis, and disrupting of mitochondrial membrane potential. Numerous clinical studies on CLAG-based regimens associated with R/R AML patients group are constantly ongoing. Based on the outcomes, the summary of our research is shown below: Firstly, the pooled CR rate, ORR, ED rate in our study are 50%, 64%, 9%, however significant heterogeneity existed within groups. Secondly, the median OS of our patients ranged from 7.3 to 14 months through a descriptive systematic review. Thirdly, the source of heterogeneity may result from treatment protocol, disease status, and risk stratification factors. The subgroup analysis of treatment protocol demonstrated that there was overlap in confidence interval for pooled CR rate between CLAG, CLAG-M, CLAG-other drugs regimens, and it is similar situation for disease status subgroup analysis, which indicated difference in results and the clinical trail redesign is needed validated. A subgroup analysis of different cytogenetic risk classification shown that significantly higher pooled CR rate in good risk group, and there was no overlapping confidence interval between good and intermidate/poor risk group which suggested that the result was no difference and stable.

There was also a meta-analysis published by Zhou et al^[28]^ that described the efficacy and toxicity of cladribine and CLAG-based treatment protocol. The outcomes proved that CLAG-based regimen is a much better choice than cladribine monotherapy, which also ascertains the effectiveness of CLAG based protocol. Their CR rate of standard CLAG chemotherapy containing 4 studies was 54.8% (95% CI: 46.2–63.1) with low heterogeneity which was lower compared with our results. Moreover, Zhou et al’s meta-analysis only included 7 records on the CLAG regimen and some of those eligible publications were somehow out of date. The number of our eligible studies is rather sufficient and simultaneously size of population group was enough to analyze, which we can overcome the shortcoming result from insufficient data.

Wierzbowska et al^[14]^ conducted a multi-center phase II study and found that 58% of R/R AML patients achieved CR after 1 or 2 CLAG-M induction chemotherapy. And Jaglal et al^[29]^ also conducted a study comparing CLAG-M therapy with standard induction therapy on AML patients with antecedent MDS after hypomethylating agents failure, superiority in improving complete response and overall survival was reported. Przespolewski et al^[30]^ compared 49 patients for CLAG-M treatment with 14 patients for CLAG treatment. Results showed the superiority of CLAG-M regimen in increasing CR and CRi rate and lower ED rate. On the one hand, these results confirmed the benefits of CLAG-M therapy, on the other hand, the divergence between these studies and our study was need a more delicate and scientific trails to explore. The numerically superiority presented with CLAG regimen for R/R AML patients in our study perhaps attributed to the relatively higher ED rate in CLAG combined with other drugs, particularly imatinib combination.

Because of limited data retrieved could not permit further analysis of the significance of efficacy and safety between other different combination drugs, like IM, aclarubicin, and seliniexor. Imatinib is a targeted drug for chronic myeloid leukemia (CML). Imatinib was proved effective in c-kit positive relapsed or refractory AML although only 4 of 12 patients achieved a hematologic remission.^[31]^ Based on the synergy of imatinib and some chemotherapeutic drugs in in vitro experiments, there was a phase I study published in 2008 that recorded an overall response rate of 43.8% for CLAG-imatinib treated patients. The median overall survival was 175 days (95% CI: 16.24–333.76). However, the sample size of this study was relatively few containing only 16 evaluable patients.^[32]^ Later, the phase II study published in 2017, which was also included in our meta-analysis, enrolled 38 patients; the results showed the ORR is 37%. However, further study is still needed to verify our findings.

In the nature or non random controlled clinical trails is inevitable, and the probable bias within group or between groups was nor very clear to explicit, further exploration regarding CLAG in R/R AML patients is emergent. But since the clinical truth is usually giving up further treatment when confronting the relapsed/ refractory setting especially avoiding suffering for some old people, so the process of such clinical trails is rather complicated than we thought.

## 5. Conclusion

In conclusion, the benefit of the CLAG regimen for improving response rate and survival of R/R AML patients is positive, and the CLAG-M treatment protocol does have potential benefit. However, there is no commonly accepted standard first-line salvage therapy protocol for R/R AML. Thus, there is still the need to conduct more RCTs to provided more important and quality statistical proof for other combinations with the CLAG regimen.

## Author contributions

**Conceptualization:** Susu Cao, Yi Dong.

**Data curation:** Susu Cao, Qianshan Tao, Jia Wang, Qing Zhang, Yi Dong.

**Formal analysis:** Susu Cao, Jia Wang.

**Methodology:** Susu Cao.

**Supervision:** Qing Zhang.

**Writing – original draft:** Susu Cao.

**Writing – review & editing:** Qianshan Tao, Jia Wang, Qing Zhang, Yi Dong.

## Supplementary Material
