# Supplementary material for: Cladribine, cytarabine, and filgrastim based regimen in relapsed or refractory acute myeloid leukemia: A systematic review and meta-analysis
Source: Medicine (Baltimore). 2023 Nov 3;102(44):e34949. doi: 10.1097/MD.0000000000034949 (PMC10627662; doi:10.1097/MD.0000000000034949)
Supplement: Supplementary file 1 [file medi-102-e34949-s001.doc]

| **Study** | **selection** | | | | **Comparability** | **Outcome** | | | **Score** |
| --- | --- | --- | --- | --- | --- | --- | --- | --- | --- |
|  | **Representativeness of the exposed cohort** | **Selection of the non exposed cohort** | **Ascertainment of exposure** | **Demonstration that outcome of interest was not present at start of study** | **Comparability of cohorts on the basis of the design or analysis** | **Assessment of outcome** | **Was follow-up long enough for outcomes to occur** | **Adequacy of follow up of cohorts** |  |
| **Bao, et al. [10]** | ★ | ★ | ★ | ★ | ★★ | ★ | ★ | ★ | 9 |
| **Duan, et al.[11]** | ★ | ★ | ★ | - | - | ★ | ★ | ★ | 6 |
| **Mirza, et al.[12]** | ★ | - | ★ | ★ | - | ★ | ★ | ★ | 6 |
| **Wang, et al. [13]** | ★ | ★ | ★ | ★ | - | ★ | ★ | ★ | 7 |
| **Wierzbowska, et al.[14]** | ★ | ★ | ★ | ★ | - | ★ | ★ | ★ | 7 |
| **Wrzesien´-Kus, et al. [15]** | ★ | - | ★ | ★ | - | ★ | ★ | ★ | 6 |
| **Park, et al. [16]** | ★ | ★ | ★ | - | ★★ | ★ | ★ | ★ | 8 |
| **Price, et al. [17]** | ★ | ★ | ★ | - | ★★ | ★ | ★ | ★ | 8 |
| **Xu, et al. [18]** | ★ | ★ | ★ | - | - | ★ | ★ | - | 5 |
| **Mushtaq, et al. [19]** | ★ | ★ | ★ | - | ★★ | ★ | ★ | ★ | 8 |
| **Patzke, et al.[20]** | ★ | ★ | ★ | - | ★★ | ★ | ★ | ★ | 8 |
| **Ye, et al.[21]** | ★ | ★ | ★ | - | - | ★ | ★ | ★ | 6 |
| **Wang, et al.[22]** | ★ | ★ | ★ | - | - | ★ | ★ | ★ | 6 |
| **Abboud, et al.[23]** | ★ | - | ★ | ★ | - | ★ | ★ | ★ | 6 |
| **Halpern, et al.[24]** | ★ | ★ | ★ | ★ | ★★ | ★ | ★ | ★ | 9 |

**Supplementary Table1.** The quality of the included studies assessed by NOS.
