# Supplementary figures and images for: Cladribine, cytarabine, and filgrastim based regimen in relapsed or refractory acute myeloid leukemia: A systematic review and meta-analysis
Source: Medicine (Baltimore). 2023 Nov 3;102(44):e34949. doi: 10.1097/MD.0000000000034949 (PMC10627662; doi:10.1097/MD.0000000000034949)

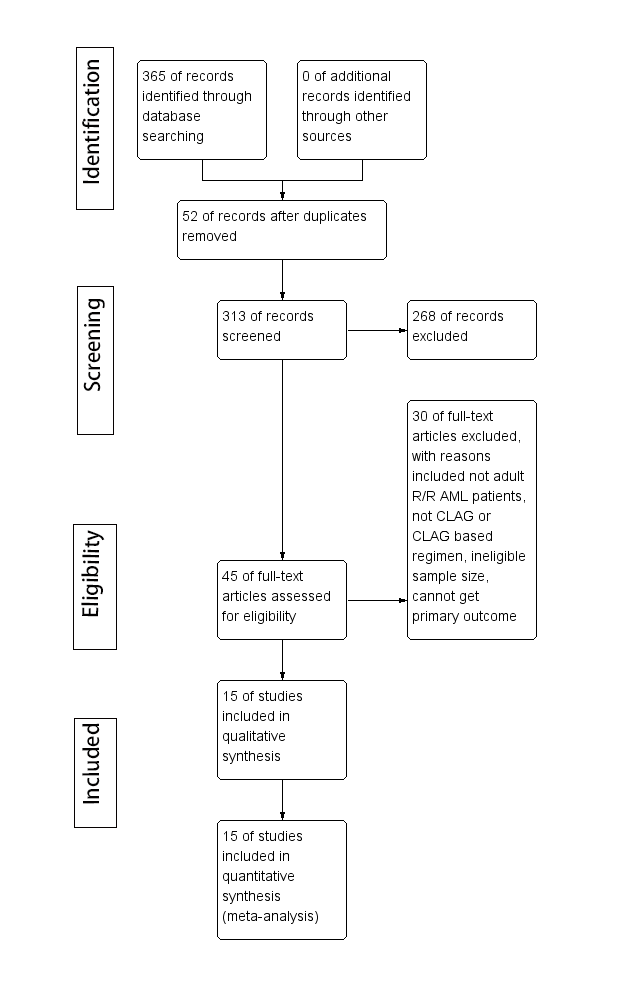

Supplement: Supplementary file 2 [file medi-102-e34949-s002.tif]

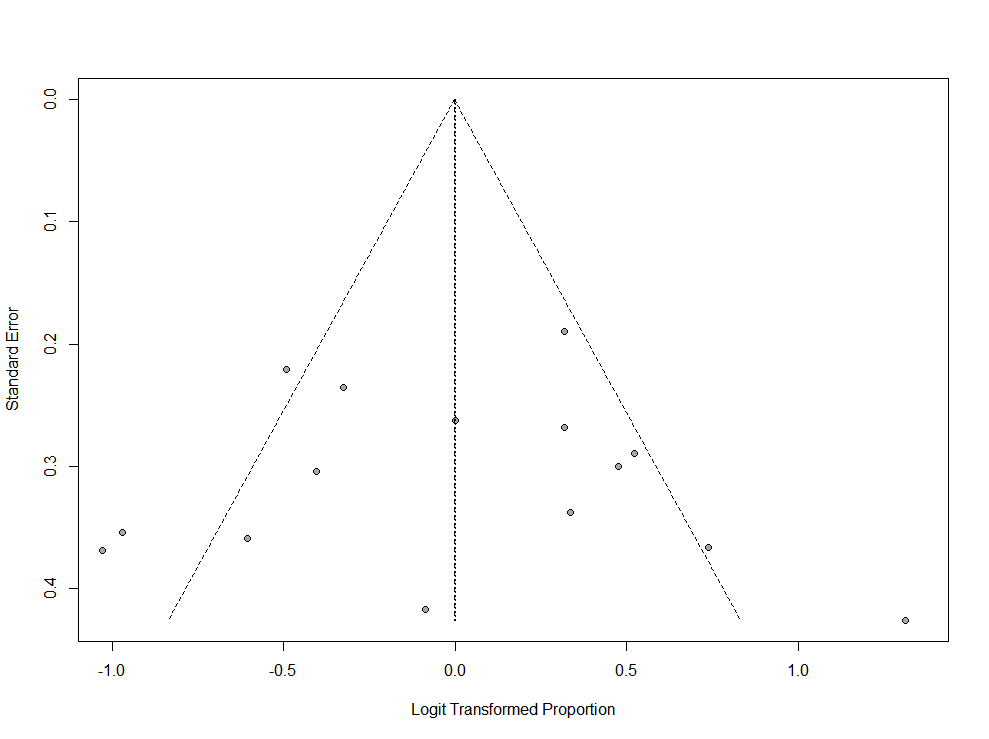

Supplement: Supplementary file 4 [file medi-102-e34949-s004.tif]

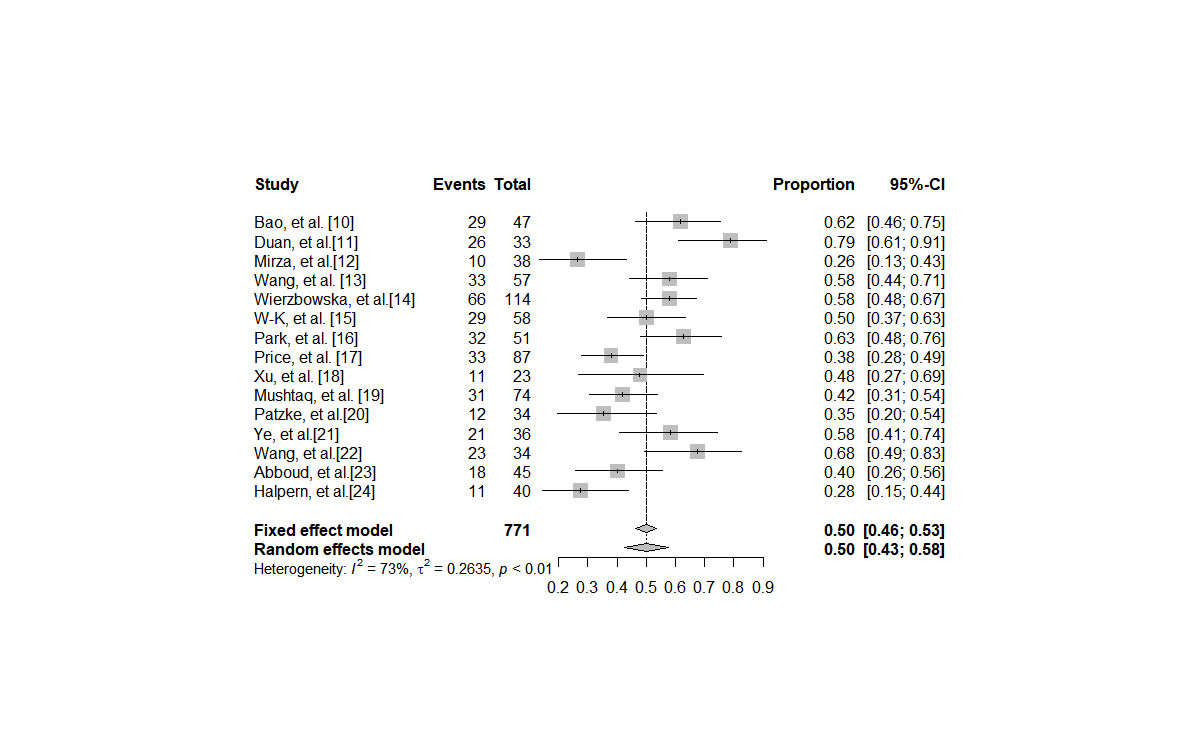

Supplement: Supplementary file 5 [file medi-102-e34949-s005.tif]

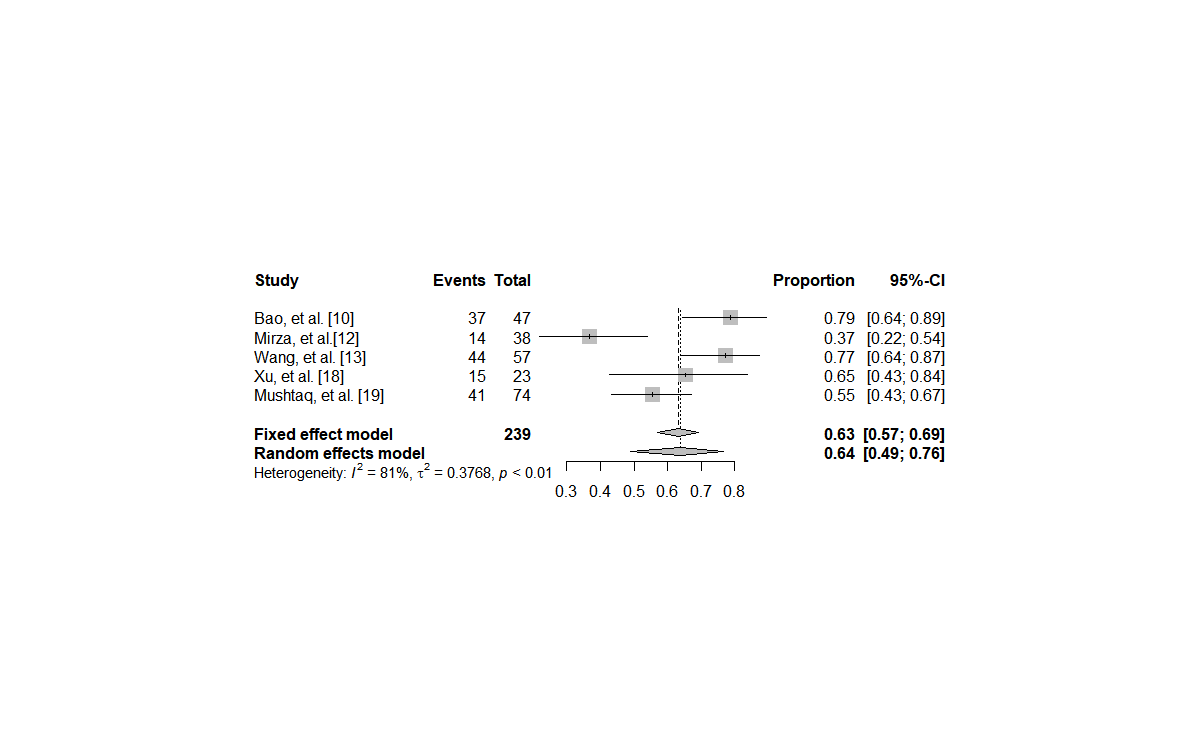

Supplement: Supplementary file 6 [file medi-102-e34949-s006.tif]

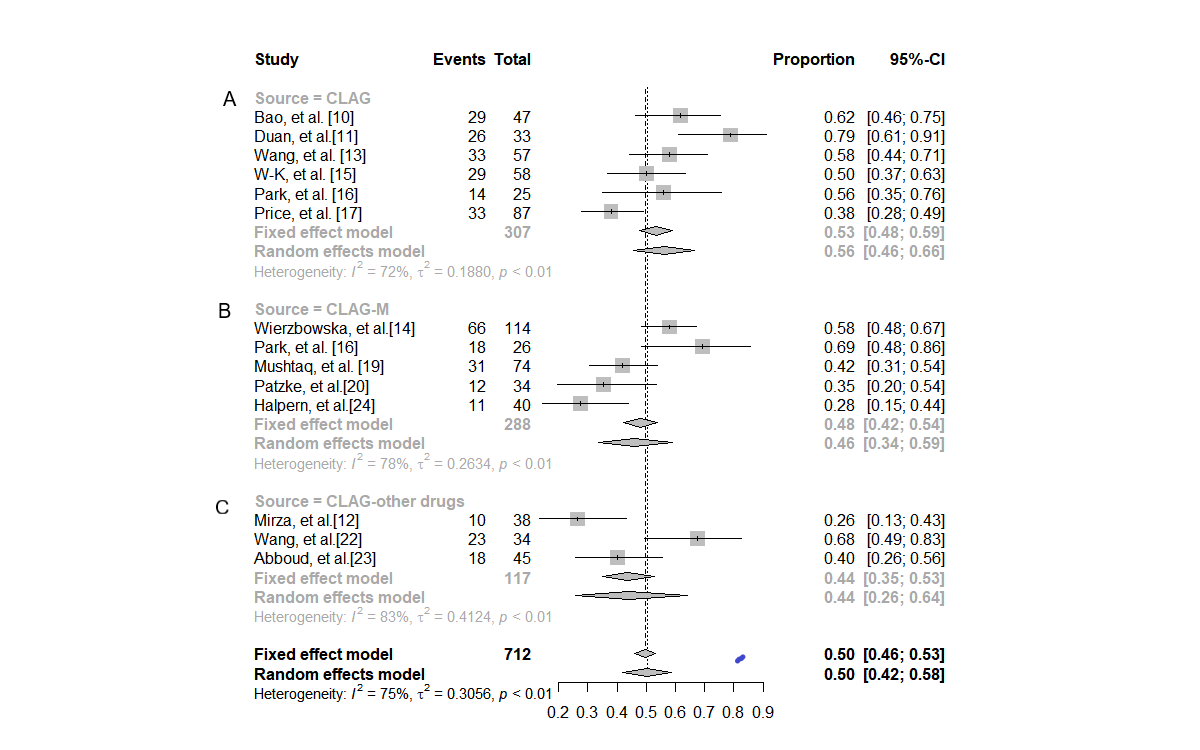

Supplement: Supplementary file 7 [file medi-102-e34949-s007.tif]

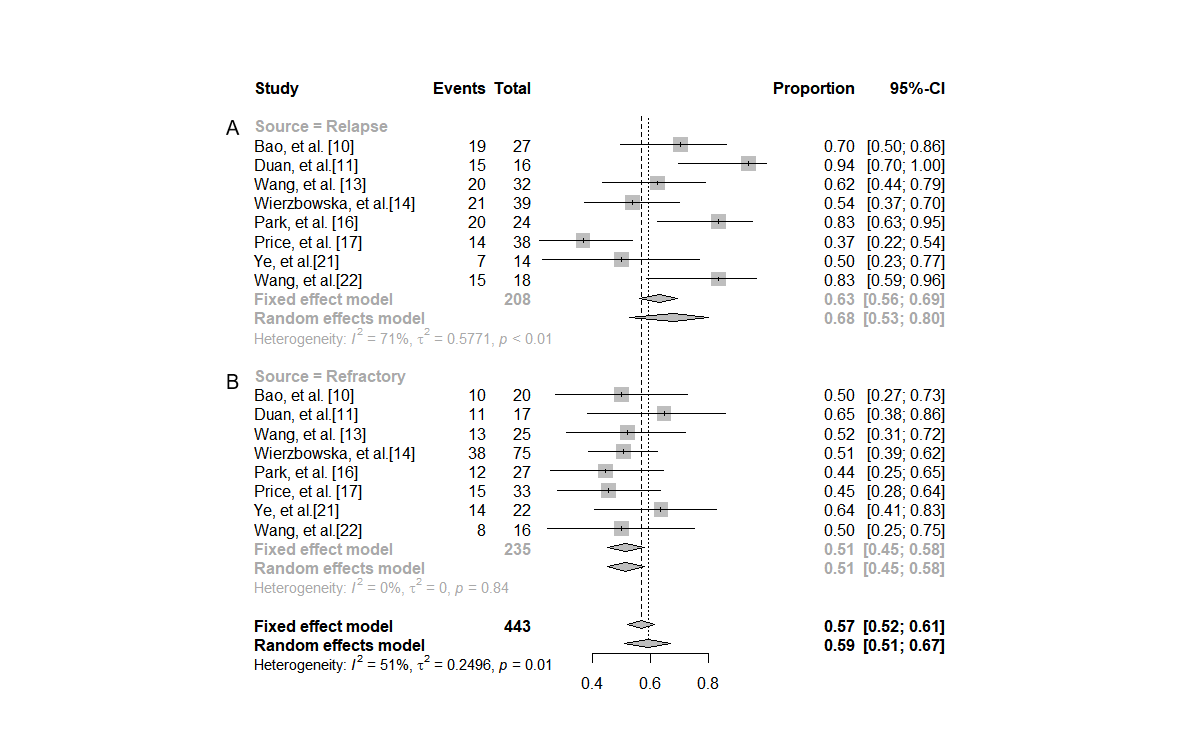

Supplement: Supplementary file 8 [file medi-102-e34949-s008.tif]

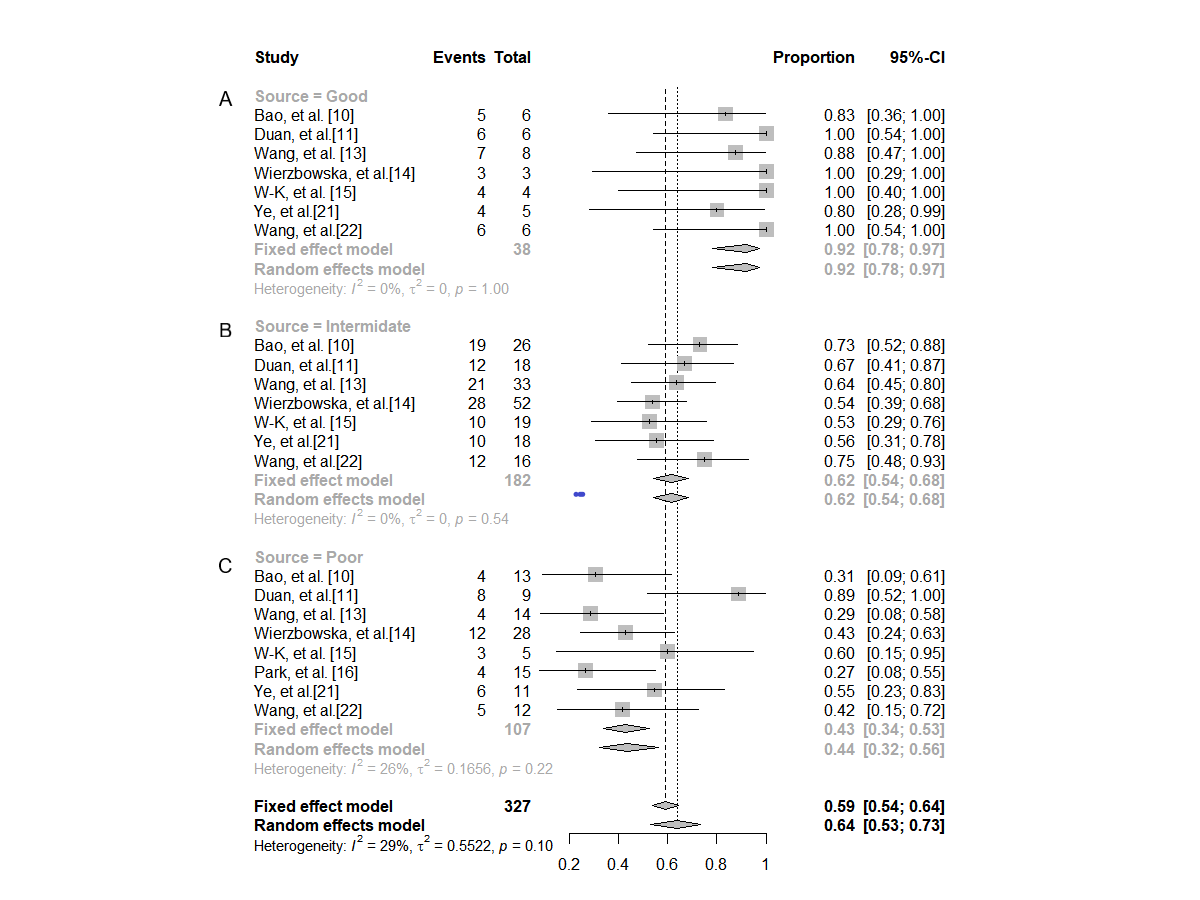

Supplement: Supplementary file 9 [file medi-102-e34949-s009.tif]

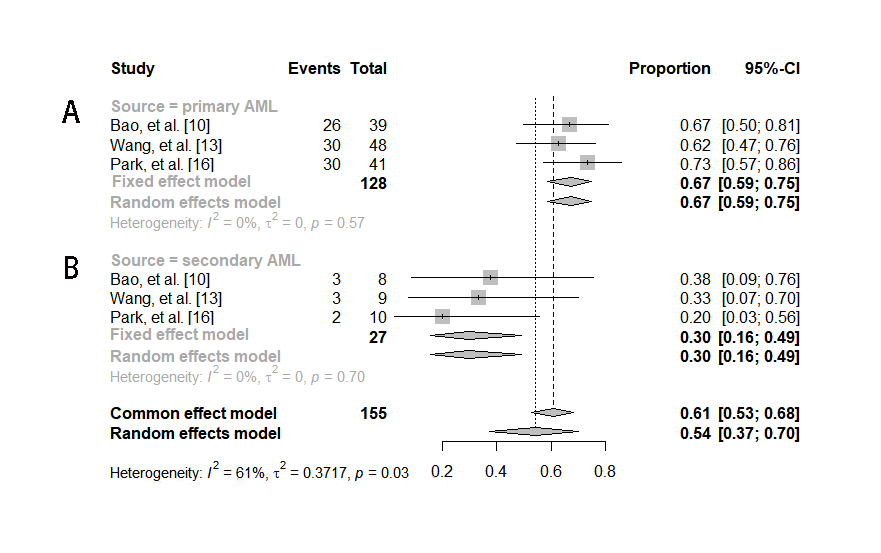

Supplement: Supplementary file 10 [file medi-102-e34949-s010.jpg]

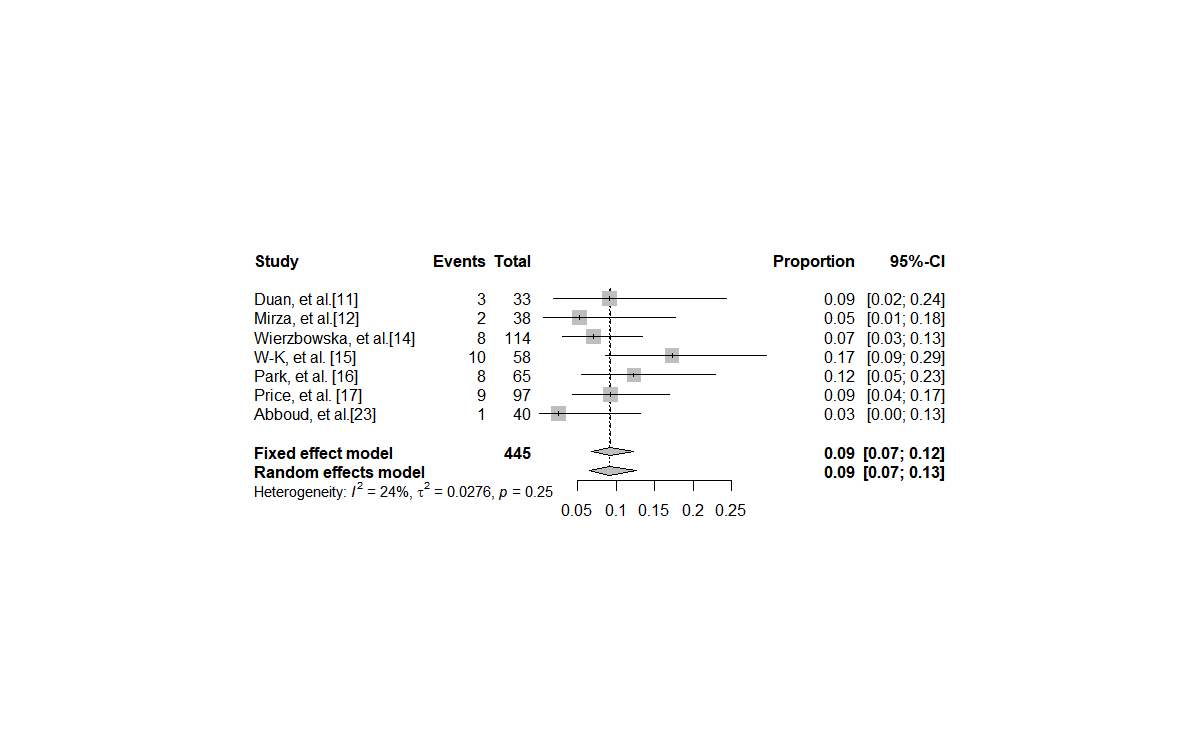

Supplement: Supplementary file 11 [file medi-102-e34949-s011.jpg]
