# Supplementary material for: Cladribine, cytarabine, and filgrastim based regimen in relapsed or refractory acute myeloid leukemia: A systematic review and meta-analysis
Source: Medicine (Baltimore). 2023 Nov 3;102(44):e34949. doi: 10.1097/MD.0000000000034949 (PMC10627662; doi:10.1097/MD.0000000000034949)
Supplement: Supplementary file 3 [file medi-102-e34949-s003.doc]

**Supplementary Table 2. Basic characteristics of included studies.**

| **Study** | **Year** | **Disease** | **Induction regime** | **Number of patients analyzed** | **Median age (years)** | **Gender(m)** | **Risk classification (good; intermidate; poor; NK; NA)** | **BM blasts(%)** | **CR (%)** | **ORR (%)** | **OS(m)** | **DFS(m)** | **ED (%)** |
| --- | --- | --- | --- | --- | --- | --- | --- | --- | --- | --- | --- | --- | --- |
| **Bao, et al. [10]** | 2017 | R/RAML | CLAG cladribine5mg/m2/dayd1–5,cytarabine2g/m2/ dayd1–5,filgrastim 300µg/dayd0–5 | 55 | 51.0±17.7 | 31 | 6;30;17 | 43.2(25.1–61.8) | 61.7 | 78.7 | 12.0(95%CI8.4–15.6) | NA | NA |
| **Duan, et al.[11]** | 2016 | R/RAML | CLAG cladribine5mg/m2/dayd1–5,cytarabine2g/m2/ dayd1–5,filgrastim 300µg/dayd0–5 | 33 | 49（14-68） | 16 | 6;18;9 | 29 （55-91） | 78.8 | NA | 14.0（0.3-17.5） | NA | 9.1 |
| **Mirza, et al.[12]** | 2017 | R/RAML | CLAG+IM cladribine5mg/m2/dayd1–5,cytarabine2g/m2/ dayd1–5,filgrastim 300µg/dayd0–5,IM400mgbidd2-15;Oneortwocycles | 38 | 62 (26-79) | 22 | 2；18；16 | NA | 26 | 38 | 11.1 (95%CI,4.8-13.4) | NA | 5.3 |
| **Wang, et al. [13]** | 2018 | R/RAML | CLAG cladribine5mg/m2/dayd1–5,cytarabine2g/m2/ dayd1–5,filgrastim 300µg/dayd0–5 | 67 | 50.54±17.75 | 36 | 8;36;21;2 | 42.1(30.1–65.0) | 57.9 | 77.2 | 10.0(95%Cl7.9–12.1) | NA | NA |
| **Wierzbowska, et al.[14]** | 2007 | R/RAML | CLAGM cladribine 5mg⁄m2/dayd1-5,cytarabine2g⁄m2/dayd1-5,MIT10mg⁄m2d1-3,G-CSF300µg/dayd0-5;Oneortwocycles | 114 | 45(20–66) | 61 | 3;52;28;12;19 | 44(6–97) | 58 | NA | 9 | 17 | 7 |
| **Wrzesien´-Kus, et al. [15]** | 2003 | R/RAML | CLAG cladribine5mg/m2/dayd1–5,cytarabine2g/m2/ dayd1–5,filgrastim 300µg/dayd0–5;Oneortwocycles | 58 | 45 (18–67) | 30 | 4;19;5;4;26 | 77(30–100) | 50 | NA | 8.5(0.25– 51.5+) | 4.25(0.25– 50.5+) | 17 |
| **Park, et al. [16]** | 2016 | R/RAML | CLAG/CLAGMCLAG cladribine5mg/m2d1–5,cytarabine2g/m2d1– 5,filgrastim300μgd0–4;CLAGM:theprevioustreatmentsplus10mg/m2mitoxantrone d1-3 | 65 | 54.0(19–83) | 34 | 10;35;19;1 | 62.6(7.6–96.1) | 62.7 CLAG56 CLAGM69.2 | NA | 13.3 | NA | 12.3±4.1 |
| **Price, et al. [17]** | 2010 | R/RAML | CLAG cladribine5mg/m2/dayd2–6,cytarabine 2g/m2/dayd2–6,filgrastim300µg/dayd1–6 | 97 | 55.1(23–83) | 62 | 4;53;30;10 | 58.1(12–97) | 37.9 | NA | 7.3 | NA | 9.40 |
| **Xu, et al. [18]** | 2018 | R/RAML | CLAG-based cladribine5mg/m2/dayd1–5,cytarabine2g/m2/ dayd1–5,filgrastim 300µg/dayd0–5,withorwithoutmitoxantrone10mg/m2/day d1–3 | 27 | NA | NA | NA | NA | 47.8 | 65.2 | 10.0(95% CI3.4–16.6) | NA | NA |
| **Mushtaq, et al. [19]** | 2020 | R/RAML | CLAG-M cladribine5mg/m2,cytarabine2g/m2,andfilgrastim300mcgfor5dayswithmitoxantrone10mg/m2for3days | 74 | 60(23–77) | 41 | 16;22;36;13 | 30 | 42 | 55 | 13.395%CI2.4–24.3 | NA | NA |
| **Patzke, et al.[20]** | 2020 | R/RAML | CLAG-M cladribine5mg/m2/dayd1–5,cytarabine2g/m2/ dayd1–5,filgrastim 300µg/dayd0–5，mitoxantrone 10mg/m2d1-3 | 34 | 52(20–67) | 36 | NA | 40(5–88) | 35.3 | NA | NA | NA | NA |
| **Ye, et al.[21]** | 2019 | R/RAML | ModifiedCLAG cladribine5mg/m2/dayd1-5,cytarabine 1g/m2/dayd1-5,G-CSF 5μg/kgbeginningthedaybeforechemotherapyandcontinu- ingdailyuntilneutrophilrecovery,oneortwocycles | 36 | 45.8(21.5–67.9 | 20 | 5;18;11;2 | NA | 58 | NA | 10.8(95%CI,6.0–15.7) | NA | NA |
| **Wang, et al.[22]** | 2020 | R/RAML | C-CAG cladribine5mg/m2,/dayd1–5; G-CSF300μg/dayd0–9;aclarubicin10mg,d3–6; cytarabine10mg/m2every12hoursd 3–9;4weekspercycle;oneortwocycles | 34 | 47(18–72) | 19 | 6;16;12 | NA | 67.6 | NA | NA | NA | NA |
| **Abboud, et al.[23]** | 2019 | R/RAML | CLAG+seliniexor selinexor60mg/dayorallyd1,5,10,12,cladribine5mg/m2/dayd4-8,G-CSF300mcgSConceperdayond3-8,cytarabine2g/m2/dayd4-8. | 40 | 55.5(21-70) | 25 | 2；27；9；2 | NA | 45 | NA | 7.8 (95%CI5.7-14.1) | NA | 2.5 |
| **Halpern, et al.[24]** | 2018 | R/RAML | CLAG-M mitoxantrone16mg/m2/dayd1-3,G-CSF d0-5,cladribine5 mg/m2/dayd1-5,cytarabine2g/m2/dayd1-5. Thefirst2dosesofG-CSFcouldbeomittedifthetotal whitebloodcellcountwas>20,000/μL | 40 | 63(33-77) | 24 | NA;25;15 | NA | 28 | NA | 11 | NA | NA |

Abbreviations: NA, Not available.
